# Intrinsic and adaptive myelination—A sequential mechanism for smart wiring in the brain

**DOI:** 10.1002/dneu.22518

**Published:** 2017-09-14

**Authors:** Marie E. Bechler, Matthew Swire, Charles ffrench‐Constant

**Affiliations:** ^1^ MRC Centre for Regenerative Medicine and MS Society Edinburgh Centre for MS Research, The University of Edinburgh, 5 Little France Drive Edinburgh EH16 4UU United Kingdom

**Keywords:** myelin, oligodendrocyte, plasticity, activity‐dependent, glia

## Abstract

The concept of adaptive myelination—myelin plasticity regulated by activity—is an important advance for the field. What signals set up the adaptable pattern in the first place? Here we review work that demonstrates an intrinsic pathway within oligodendrocytes requiring only an axon‐shaped substrate to generate multilayered and compacted myelin sheaths of a physiological length. Based on this, we discuss a model we proposed in 2015 which argues that myelination has two phases—intrinsic and then adaptive—which together generate “smart wiring,” in which active axons become more myelinated. This model explains why prior studies have failed to identify a signal necessary for central nervous system myelination and argues that myelination, like synapses, might contribute to learning by the activity‐dependent modification of an initially hard‐wired pattern. © 2017 The Authors. Developmental Neurobiology Published by Wiley Periodicals, Inc. Develop Neurobiol 78: 68–79, 2018

## INTRODUCTION: THE CONCEPT OF ADAPTIVE MYELINATION

Myelin‐forming oligodendrocytes are found in both white and gray matter of the central nervous system (CNS), and the internodes or sheaths they form around axons represent a spectacular cell–cell interaction that enables rapid saltatory conduction. This is facilitated by the concentration of voltage‐dependent sodium channels at the gap between sheaths—the nodes of Ranvier. The prevailing view since the discovery of this mechanism in 1949 has been that both internode length and thickness are optimized simply for maximum conduction velocity and the reduction of energy consumption (Huxley and Stämpfli, 1949; Rushton, 1951). Recently, however, a number of observations challenge this view and point to additional roles of oligodendrocytes and myelination in a fundamental property of the CNS—to modify circuits in response to experience. First, new oligodendrocytes are generated throughout life from actively dividing precursor cells (Rivers et al., 2008; Hughes et al., 2013; Young et al., 2013), and formation of these newly differentiated cells improves motor learning (McKenzie et al., 2014; Xiao et al., 2016). Second, myelination in the cortex is reduced by social isolation (Liu et al., 2012; Makinodan et al., 2012), and the associated social avoidance behavior can be rescued by the muscarinic antagonist clemastine that increases oligodendrocyte differentiation (Liu et al., 2016). Third, increasing activity using optogenetics enhances oligodendrocyte precursor cell proliferation, myelination, and improves motor performance (Gibson et al., 2014). Fourth, myelination of pyramidal projection neuron axons in the cortex is discontinuous, suggesting a mechanism by which the myelin formed by newly generated oligodendrocytes could alter axonal conduction velocities (Tomassy et al., 2014). Last, magnetic resonance imaging studies of adult humans learning complex motor tasks show changes in associated axonal tracts consistent with increased myelination (Scholz et al., 2009; Sampaio‐Baptista et al., 2013). Together, these observations have led to the concept of adaptive myelination—changes in oligodendrocytes and the myelin they form in response to activity enhance conduction and so reinforce active pathways. They do not, however, explain how the adaptable pattern of myelination is generated in the first place. Here, by reviewing relevant literature on myelin sheath formation and plasticity, we address this question and develop a model in which sequential intrinsic and adaptive pathways create “smart” wiring that changes in response to activity and which, alongside synaptic plasticity, enables learning.

## THE UNSUCCESSFUL SEARCH FOR AXONAL SIGNALS NECESSARY FOR CNS MYELINATION

A prediction of the adaptive myelination hypothesis is that, as is implicit in the name, a prior pattern of myelin is created by an independent set of pathways and signals. Based on work in the peripheral nervous system (PNS), where the level of neuregulin (NRG) 1 type III expression on the axon determines both whether the axon is myelinated by Schwann cells and the thickness of the sheath formed (Michailov et al., 2004; Taveggia et al., 2005), much attention has focused on the identification of axonal signals in the CNS. However, despite more than a decade of study, no such signals have been found. Although overexpression of NRG in neurons does increase myelin sheath thickness, showing that oligodendrocytes (alike Schwann cells) can respond to NRG signaling by increasing myelination, conditional knockout mice (cKO) lacking NRG 1 in neurons, or loss of all NRG signaling by double cKO of ErbB3 and ErbB4 receptors in oligodendrocytes, show NRG signaling is not required for the establishment of myelination in the CNS (Brinkmann et al., 2008). Extracellular matrix receptors of the integrin family contribute to the initiation of myelination but again are not required for normal white matter myelination (Benninger et al., 2006; Câmara et al., 2009). Finally, NMDA glutamate receptors localized in the myelin sheath (Micu et al., 2006, 2016; Saab et al., 2016) can enhance myelination in cell culture (Wake et al., 2011; Lundgaard et al., 2013) yet are dispensable for in vivo myelination as revealed by oligodendrocyte‐specific cKO mice (De Biase et al., 2011). Together, these experiments show that these signals are not required for the initial formation of myelin sheaths although—as we will discuss below—they do not exclude important roles in subsequent adaptive changes.

## AN INTRINSIC MYELINATION PROGRAM REQUIRES ONLY AN APPROPRIATE PHYSICAL SUBSTRATE FOR MYELIN SHEATH FORMATION

Given the lack of any known necessary instructive molecules, an alternative hypothesis that oligodendrocytes have intrinsically encoded information to carry out myelin sheath formation, not requiring extrinsic molecular instruction, warrants consideration. The notion of an intrinsically encoded program for oligodendrocyte precursor cells' development into mature cells is not new. It was shown decades ago that in the absence of neuronal instruction, oligodendrocytes have an intrinsic timing program for differentiation and the generation of specialized membranes of similar molecular composition to myelin (Mirsky et al., 1980; Szuchet et al., 1983; Dubois‐Dalcq et al., 1986; Gard and Pfeiffer, 1989). These flat‐membrane monolayers generated by oligodendrocytes in culture do not, however, recapitulate the complex 3D architecture of myelin sheaths in vivo, raising the question as to whether the signals required for this architecture can also be driven by an intrinsic program or whether signals from the axon are required for this final stage of myelination. To address this, a number of groups have investigated oligodendrocytes in neuron‐free 3D cultures. Initial studies demonstrated the capacity of oligodendrocytes to form an initial layer of membrane wrapping around axon‐sized, cylindrically shaped substrates of carbon, glass, and lactide/glycoside copolymers, and vertical compressed silica micropillars (Althaus et al., 1987; Bullock and Rome, 1990; Howe, 2006; Mei et al., 2014). These investigations did not, however, demonstrate the formation of internode‐like structures that spirally wrap around axons in numerous membrane bilayers comparable to those found in vivo. While electron microscopy demonstrated that multilamellar compact membranes could form on chemically fixed axons, indicating that active cues from neurons are not essential (Rosenberg et al., 2008), cross‐linked axonal cell surface molecules were present, still leaving the possibility that cell–cell adhesion molecules or other membrane components instruct myelin formation. Seminal progress has been the recent development of neuron‐free, electrospun microfiber cultures, demonstrating the capacity for oligodendrocytes to generate compact, multilamellar myelin membranes ensheathing microfibers (Lee et al., 2012a, 2013; Bechler et al., 2015). These data finally provide conclusive evidence that oligodendrocytes, in the presence of appropriate physical cues, do contain the encoded information to generate the proper three‐dimensional architecture of myelin; in other words, that myelin sheath formation is driven by an intrinsic pathway.

## THE INTRINSIC MYELINATION PROGRAM GENERATES MYELIN SHEATHS OF PHYSIOLOGICAL LENGTH

What about the conduction velocity‐altering properties—placement, length, and thickness—of these myelin sheaths; are these under axonal control or does an intrinsic program also regulate these properties? Surprisingly, we found that cortical oligodendrocytes in microfiber cultures generated myelin sheath lengths equivalent to that on dorsal root ganglia neurons of comparable diameters and similar to prior reports of myelin sheath lengths in the mouse cortex (Murtie et al., 2007; Bechler et al., 2015). While each oligodendrocyte formed significantly fewer sheaths than can be found in vivo, this raises the intriguing possibility that at least the fundamental property of myelin sheath length is set prior to oligodendrocyte precursor differentiation. However, would that be consistent with the heterogeneity of myelin sheath distribution and sizes seen across the CNS (del Río‐Hortega, 1928; Hildebrand et al., 1993)? If oligodendrocyte precursor cells, upon initiating differentiation, do not require extrinsic molecular instruction to form myelin sheaths with similar properties to that found on neurons, how is myelin sheath variation achieved in the CNS? Current work supports two possible intrinsic mechanisms to instruct myelin sheath size: oligodendrocytes sense and respond to the physical size of axons, and oligodendrocyte populations exist with heterogeneous intrinsic programs. The evidence for and predictions of each are discussed below.

## PHYSICAL CUES DICTATE MYELIN SHEATH LENGTH

Axon caliber has been a key property proposed to correlate with myelin sheath length and thickness. Numerous anatomical studies, including those of spinal cord white matter in a variety of vertebrates, have documented the relationship of axon diameter with the associated myelin sheath length and thickness, demonstrating a positive correlation (e.g., Duncan, 1934; Hildebrand and Hahn, 1978; also reviewed in Hildebrand et al., 1993). Thickness (number of lamellae) of myelin sheaths has been stereotyped for axon diameters by the so‐called g‐ratio (ratio of axon diameter to the outer diameter of the myelin sheath) averaging around 0.7. Further studies also indicate that internode length increases with axonal diameter (McDonald and Ohlrich, 1971; Murray and Blakemore, 1980; Ibrahim et al., 1995; Butt et al., 1998). While axonal molecules could increase with larger axon calibers, our fiber experiments show that the physical diameter of the axons itself may serve as a signal that determines myelin sheath length. Isolated rodent oligodendrocytes show a remarkable response of increasing myelin sheath length in accordance to different diameter microfibers in the absence of additional signals (Bechler et al., 2015). While it remains to be determined if the number of myelin layers (lamellae) are similarly adjusted purely by physical fiber size, this work suggests that oligodendrocytes have curvature‐sensitive mechanisms to regulate the lateral expansion of myelin membranes along a microfiber (or axon). However, while many studies have shown a linear correlation between axon caliber and internode length or thickness, as a simple curvature‐sensing model would predict, exceptions to this rule exist. The smallest and largest caliber axons measured (<1 micron and >8 micron diameters), exhibit nonlinear changes in sheath length or thickness (Hildebrand and Hahn, 1978). For thicker axons, this might be explained by the fact that, once above a certain threshold diameter size, the change in curvature sensed by an oligodendrocyte is unlikely to be significant. One might therefore expect a plateau where increasing sheath size will be subtle in comparison to those seen with smaller caliber axons. This would not, however, explain the loss of correlation on thinner axons. Additionally, myelin sheath lengths become steadily shorter along auditory pathway neurons as they approach the axonal terminal synapsing onto the giant Calyx of Held (Ford et al., 2015). These rule‐defying examples showing variance in both lengths and thickness are indicative that other factors beyond physical cues further instruct or fine‐tune sheath sizes.

## INTRINSIC PROPERTIES OF HETEROGENEOUS OLIGODENDROCYTES MAY DETERMINE MYELIN SHEATH LENGTH

The presence of heterogeneously programed oligodendrocyte populations is another possible contributor to CNS myelin sheath variation. Direct evidence for oligodendrocyte heterogeneity has been demonstrated with genetic lineage tracing and by molecular profiling. Lineage tracing studies have demonstrated the presence of dorsal and ventral oligodendrocyte precursor cell populations arising from distinct subventricular zones at different times during mouse development (Kessaris et al., 2006; Tripathi et al., 2011; also see reviews: Richardson et al., 2006; Rowitch and Kriegstein, 2010). To address whether these populations compensate for each other (i.e., are interchangeable) or were destined to distinct fates, ablation of each population was conducted. In the absence of each population, the reciprocal pool of OPCs repopulated the CNS regions and gross myelination was unperturbed (Kessaris et al., 2006), indicating that the cell populations can compensate for the loss of the others to a certain extent. Whether these populations maintain an independent identity, epigenetic program, or have functional differences has yet to be demonstrated. On a transcriptional level, recent single‐cell RNA sequencing of the oligodendrocyte lineage in brain and spinal cord gray matter regions has shown six transcriptionally distinct clusters of oligodendrocytes present throughout the period of developmental myelination (Marques et al., 2016). It is unknown at present to what extent the six populations may represent distinct myelinating oligodendrocyte populations, a continuum of oligodendrocyte maturation, or have different transcriptional profiles merely based on local cell–cell interactions—important areas for further work.

The notion that intrinsic differences such as these between subpopulations of oligodendrocytes might instruct differing sheath profiles was raised by the studies of Pío del Río‐Hortega nearly a century ago, who used myelin sheath length and number to distinguish four types of oligodendrocytes (del Río‐Hortega, 1928; Butt et al., 1998). The hypothesis that oligodendrocyte populations are intrinsically restricted to form sheaths on axons of a particular diameter or a particular number of myelin sheaths has been tested in two ways. First, by transplantation of oligodendrocytes from rodent optic nerve, which normally myelinate relatively uniform, small caliber axons, into the spinal cord, with much larger and varied axon calibers. This demonstrated that oligodendrocytes are not tightly restricted by an intrinsic program to myelinate a select population of axons of either select caliber or function (Fanarraga et al., 1998). Second, by analyzing oligodendrocytes in genetically manipulated zebrafish that contain extra, large caliber axons. Oligodendrocytes in the zebrafish ventral spinal cord primarily generate one to three myelin sheaths on large caliber axons or many myelin sheaths on small caliber axons. Manipulation of the number of large caliber axons, however, stimulated oligodendrocytes to ensheath both the supernumerary large axons and the small diameter axons (Almeida et al., 2011).

While these experiments indicate that oligodendrocytes are not differentially programed to only myelinate either small or large caliber axons, the myelin sheaths produced by optic nerve oligodendrocytes transplanted into spinal cord were thinner than that of local spinal cord oligodendrocytes (Fanarraga et al., 1998), consistent with the existence of heterogeneity within white matter oligodendrocytes in the programs that determine sheath size. Cross‐transplantation has also been used to examine heterogeneity between adult white matter and gray matter oligodendrocytes, demonstrating that precursor populations are not equivalent in their behavior. Heterotopic and orthotopic transplantation of oligodendrocyte progenitors residing in cortical gray or white matter into the same or reciprocal region showed oligodendrocytes vary in rates of proliferation and differentiation, dependent on the site of transplantation (Viganò et al., 2013).

Together these experiments provide support for the idea that differences in oligodendrocyte morphology within the CNS reflect, at least in part, intrinsic differences in the programs that instruct sheath formation. Direct support for this comes from the isolated oligodendrocyte culture experiments with microfibers. These established that oligodendrocytes from the rodent cortex and spinal cord maintain distinct programmed behavior that determines myelin sheath lengths. Spinal cord oligodendrocytes generated much longer sheath lengths than cortical oligodendrocytes when introduced to the same physically and chemically defined environment as well as when cells were added to equivalent neuronal and ex vivo slice cultures, mirroring observations of relative sheath lengths in vivo (Bechler et al., 2015). While key questions such as when these oligodendrocyte precursor populations became intrinsically different, what the molecular differences are between these populations, and whether these differences persist throughout the age of animals remain to be addressed, this result is important in that it shows the diversity of oligodendrocyte morphology highlighted by del Río‐Hortega not only is the result of extrinsic environmental factors but also reflects intrinsic differences between the cell populations.

## INTRINSIC AND ADAPTIVE MYELINATION

**Figure 1:**
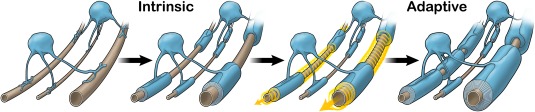
Model of intrinsic and adaptive myelination. This proposed model describes the two phases of myelination that encompass our current understanding of CNS myelination: intrinsic and adaptive. First, the intrinsic, encoded program within oligodendrocyte precursor cells guides oligodendrocyte differentiation and myelination of axons. During this intrinsic phase, oligodendrocyte processes sense physical axon diameters to select axons and generate myelin sheath lengths corresponding to the caliber, with longer sheaths on larger calibers. The second phase is adaptation of intrinsic myelin sheaths. While the timing is yet unclear, the size and number of myelin sheaths may be modified by signals from active neurons either during or after intrinsic myelination is completed. Extrinsic, adaptive signals allow for further increases to sheath size and number (as shown in the left and right axons) while no such changes occur on silent axons (middle axon). These changes may, in turn, adapt neuronal conduction and reinforce selected circuits, particularly in areas with sparsely myelinated axons. The content and concept for these images were generated by the authors and were designed and illustrated by ScideLight (www.scidelight.com). [Color figure can be viewed at wileyonlinelibrary.com]

Together with the wealth of data indicating oligodendrocyte myelination is affected by neuronal activity, these findings that oligodendrocytes only require physical dimensions to generate myelin sheaths (Lee et al., 2012a; Bechler et al., 2015) with expected sheath lengths led to our proposed model for CNS myelination: an oligodendrocyte‐intrinsic program establishes a basic pattern of myelination that can then undergo adaptation to modify myelin sheath number and/or properties (Bechler et al., 2015) (Fig. 1). As supported by microfiber cultures, this model proposes that in the absence of molecular cues from axons, oligodendrocytes will generate myelin sheaths based on a transcriptional (intrinsic) program established prior to differentiation. This transcriptionally regulated program establishes a basal sheath number and/or size. Oligodendrocyte processes could then respond locally to adapt myelin sheath number and size in response to extrinsic cues, such as those linked to activity, from the corresponding axon.

A prediction of this model is that that reduced extrinsic (adaptive) signals would still allow for myelin sheath formation, simply with a reduction in sheath size and/or number. This was initially shown over 50 years ago by Gyllensten and Malmfors (1963) who demonstrated that dark rearing mice, thus reducing the activity of retinal ganglion cells, reduced the number of myelinated axons. More recently two further sets of experiments confirm the predicted result of the model. First, real time live‐imaging studies in zebrafish have shown that silent neurons do indeed acquire myelin sheaths, and that these silent neurons in zebrafish acquire fewer, shorter sheaths than those seen on active neurons (Hines et al., 2015; Mensch et al., 2015). Second, work in mice has shown that decreasing neuronal activity within the optic nerve by monocular deprivation also does not prevent myelination but rather results in shorter myelin sheath lengths than that seen on active neurons, reducing conduction velocity (Etxeberria et al., 2016). Interestingly, the influence of adaptive signals on myelin sheaths formed only appears to occur on a subset of neuronal subtypes (Koudelka et al., 2016). Therefore, heterogeneity from neuronal subtypes may provide variation in the relative contribution of adaptive signals that influence myelination.

This model, although speculative, has value in that it reconciles current experimental data on fiber experiments, on the axonal signals involved in myelination, and on adaptive myelination. It also provides a conceptual framework for thinking about myelination in a similar way to synapse formation and plasticity, with an initially hard‐wired pattern being modified by experience so as to enable CNS plasticity and learning—in other words to create smart wiring.

An important prediction from this model is that studies seeking an axonal signal necessary for myelination will fail, as there are no such signals other than the appropriately sized physical substrate. These studies therefore now need to be re‐evaluated asking not whether the molecule under study instructs myelination but whether it contributes to the signals that subsequently adapt the sheaths formed by the intrinsic pathway. Additionally, the model generates four further questions that require consideration. First, what are the molecular pathways that account for intrinsic and adaptive myelination? Second, why are all axons and dendrites in the CNS not myelinated? Third, over what timescales do the intrinsic and adaptive mechanisms operate? Fourth, is the balance between the intrinsic and adaptive pathways that shape the ultimate morphology of each oligodendrocyte the same across different regions of the CNS? The re‐evaluation of signals and these questions will now be considered in turn.

## MOLECULAR COMPONENTS OF INTRINSIC AND ADAPTIVE MYELINATION

The sequential model described above predicts the presence of two distinct sets of signaling pathways: those that drive the formation of the myelin sheath and those that subsequently modify formation. The former, responsible for intrinsic myelination, will include processes essential for sheath formation such as the molecular pathways enabling transport of protein, locally translated RNA, and membrane lipids to nascent sheaths, and the cytoskeletal regulators required to direct the growth of three‐dimensional wraps around axons. Additionally, as the initiation of myelination and subsequent myelin sheath lengths have been shown to be sensitive to diameters (Lee et al., 2012a; Bechler et al., 2015), there must be curvature‐sensitive molecules that detect axon diameter, although none have been discovered to date. Whether or not additional transcription factors to those involved in initial oligodendrocyte differentiation and the expression of myelin proteins are required for the signaling pathways that drive sheath formation is unknown; thus far no transcription factors have been shown to specifically regulate the wrapping process. A careful analysis of the knockout mice already generated to study myelination may provide new insights, with the expectation being that those perturbing genes required for intrinsic myelination will prevent the initiation of myelin sheath formation without reducing prior oligodendrocyte differentiation, thus greatly diminishing the number of myelinated axons, while those required for adaptive myelination will show more subtle changes in myelin sheath number, thickness, and/or length. For example, the significant reductions in the number of myelinated axons by more than 50% reported with conditional knockout of the actin polymerizing Arp2/3 complex (Zuchero et al., 2015) point to a role in the intrinsic pathway.

Adaptive pathways will likely converge on these intrinsic mechanisms to alter the formation of nascent sheaths or the shape of existing myelin sheaths, thus some components of the pathways will be shared while others will be distinct to each, for example, the curvature‐sensing mechanisms in the intrinsic pathway. Proteomic studies of myelin have identified synaptic proteins and electrogenic channels in addition to the expected cytoskeletal and structural proteins (Ishii et al., 2009; Gopalakrishnan et al., 2012; Thakurela et al., 2016), suggesting the presence of myelin‐localized signaling proteins able to respond to changes in axonal activity as required for adaptive myelination. In this context, two receptors and their downstream pathways previously described as dispensable in the initial cKO studies may have their major role in adaptive myelination: the NMDA receptor and the NRG‐ErbB pathway. Oligodendrocyte NMDA receptors have been suggested to detect the vesicular release of glutamate by active axons (Ziak et al., 1998; Káradóttir et al., 2005; Micu et al., 2016), and blocking vesicular release either in vitro (Wake et al., 2011, 2015) or in zebrafish (Hines et al., 2015; Mensch et al., 2015) results in a reduction in MBP mRNA translation or sheath elongation/initiation, respectively. Roles for this receptor have also been suggested in myelin compaction (Micu et al., 2016) and in calibrating metabolic support provided by the oligodendrocyte to the axon in response to activity (Frühbeis et al., 2013; Saab et al., 2016). To reveal the true role of this receptor in myelination, it will be necessary to extend the studies reporting either no myelin phenotype in the NMDA receptor cKO mouse (De Biase et al., 2011) or a transient delay in optic nerve myelination (Saab et al., 2016) with experiments examining areas of the CNS such as the cortex where adaptive myelination has been demonstrated.

Although dispensable for the establishment of myelination, the NRG‐ErbB pathway has been directly linked to adaptive myelination: social experience regulates the expression of NRG, and mice expressing a dominant negative ErbB4 receptor in oligodendrocytes fail to show the changes in myelination associated with social experience (Makinodan et al., 2012). NRG signaling could be linked to activity by the NMDA receptor as, in myelinating co‐cultures, exposure to NRG switches the oligodendrocytes to an NMDA receptor‐dependent myelination pathway, with the receptor inhibitor MK801 blocking myelination (Lundgaard et al., 2013). A potential mechanism for this switch is provided by the finding that NRG downregulates expression of the inhibitory NR3 subunit of the NMDA receptor (Lundgaard et al., 2013). Further examination of the role of this subunit in myelination is required. Such a switch could explain the apparently contradictory finding that abolishing NRG signaling through the double cKO of ErbB3 and ErbB4, has no effect on myelin sheath formation in white matter, while overexpression of NRG1 type III increases myelin sheath thickness (Brinkmann et al., 2008). We speculate that in the absence of NRG, the myelination observed reflects the intrinsic pathway while a subsequent increase in NRG expression activates adaptive myelination, whereby axonal activity drives sheath thickening.

In addition to work focusing on signals previously evaluated in the context of their necessity for myelination, recent studies have identified other activity‐related signals that may adapt myelin—brain‐derived neurotropic factor (BDNF), GABA, and ATP. BDNF is secreted from cortical neurons following optogenetic stimulation (Venkatesh et al., 2015) and, similar to NRG, switches oligodendrocytes to a form of myelination dependent on NMDA receptors (Lundgaard et al., 2013). While global BDNF KO mice develop both axonal diameter retardation and hypomyelination (Cellerino et al., 1997; Djalali et al., 2005), oligodendrocyte‐specific loss of the BDNF receptor, TrkB, generates thinner myelin (Wong et al., 2013), consistent with a role in adaptive myelination. Cortical GABA‐positive interneurons, with larger axon calibers than non‐GABAergic neurons, have shorter myelin sheaths (Micheva et al., 2016), and a role for GABA in regulating this sheath length is suggested by the finding that blocking endogenous GABA in cortical slices increased myelin sheath length (Hamilton et al., 2017). DRG neuron cultures secrete ATP in an activity‐dependent manner promoting myelination (Stevens et al., 2002). Co‐culture studies have shown that this ATP stimulates astrocytic secretion of LIF, promoting myelination in response to neuronal activity (Ishibashi et al., 2006). This result also highlights an important point that a network of other signals from the nonmyelinating support cells of the CNS that respond to activity may also regulate adaptive myelination. In keeping with this, microglia express NRG (Ikawa et al., 2017) and the extent and significance of any changes in NRG expression in these and other supporting cell types requires further investigation.

## WHY ARE ALL AXONS AND DENDRITES IN THE CNS NOT MYELINATED?

If the initial formation of myelin sheaths is an intrinsic process requiring only the presence of an appropriate structure, one might expect all axons and dendrites in the CNS to be myelinated. Work from the Chan lab has identified two possible mechanisms that prevent this: size and inhibitory molecules. Using the microfiber assay, this group showed that only fibers with a diameter of more than 0.4 µm permit the formation of sheaths (Lee et al., 2012a). Taken together with our evidence that sheath lengths on larger microfibers increase with fiber diameter (Bechler et al., 2015), this shows that oligodendrocytes are able to detect the diameter of the structure to be myelinated, requiring a minimum diameter to initiate the process. The size threshold of 0.4 µm is similar to that above which myelination is observed in vivo (Duncan, 1934). Further support for a size threshold mechanism operating in vivo comes from elegant experiments from the Nave lab in which the PI3 kinase signaling inhibitor PTEN is removed from neurons. This leads to an increase in axonal diameter, whereby previously unmyelinated axons in the cerebellum increase in size and, at the same time, become myelinated (Goebbels et al., 2016). Second, the presence of inhibitors of myelination has been demonstrated on dendrites and neuronal cell bodies. By screening candidates identified from RNA sequencing, the adhesion molecule jam2 was identified as an inhibitory signal expressed in dendrites and neuronal soma (Redmond et al., 2016). The widespread presence of such inhibitors could therefore explain why myelin is restricted to axons in the CNS. The presence of inhibitory molecules on axons has also been suggested as a possible cause of the failure of remyelination, the effective regenerative process that can follow demyelination in the mammalian CNS. Here, PSA‐NCAM expressed on the surface of non‐re‐myelinated axons in multiple sclerosis lesions prevented myelination in culture (Charles et al., 2000; Charles, 2002). Both size‐ and inhibitor‐based mechanisms do not, however, explain the observation of discontinuous myelination of axons within cortical gray matter, as shown for pyramidal neuron projection axons (Tomassy et al., 2014). This intermittent pattern occurs despite the presence of abundant oligodendrocytes in gray matter. Inhibitors would not explain discontinuous myelination unless they were present in the pattern of stripes along the axon. While axons do have the ability to cluster molecules present at and around the nodes of Ranvier, there is no current evidence of a broader organization of stripes that might result in an inhibitor‐based pattern of discontinuous myelination. How this is achieved and the biological relevance of such myelin profiles remains an extremely important question for the field.

## OVER WHAT TIMESCALES DO THE INTRINSIC AND ADAPTIVE MECHANISMS OPERATE?

Our model proposes that an already established, intrinsically encoded program of myelin sheath formation can be tuned or modified by extrinsic signals. This intrinsic program is established prior to differentiation, and adaptive myelination likely occurs during or after differentiation has begun. A remaining question is therefore one of timing—are oligodendrocytes capable of adapting myelin sheath formation throughout their lifetime, or is there a critical time period when oligodendrocytes are receptive to extrinsic influences? The answer will be important, having implications for the potential roles and limitations of myelin adaptation in brain plasticity, learning, and myelin regeneration. However, the majority of the current evidence for adaptive myelination by extrinsic signals comes from a combination of in vitro and in vivo studies during the peak of developmental myelination (Wake et al., 2011; Hines et al., 2015; Mensch et al., 2015), and this focus on developing systems means the question of timing remains unresolved.

Live imaging studies and studies in older animals will be required to resolve this issue, but the current evidence supports both timescales for adaptive myelination. In support of a critical period, two weeks of social isolation for mice from postnatal age 21 to 35 affected behavior and myelin sheath number, length, and thickness, while two weeks of social isolation from postnatal day 35 did not affect behavior nor myelination (Makinodan et al., 2012). As the cortex is being actively myelinated at this time, this is consistent with the idea of adaptive influences being limited to differentiating and newly formed oligodendrocytes. A similar study by Liu et al. (2012) appears at first sight not to show such a “critical window,” as myelin sheath changes were observed with social isolation of mice for 8 weeks from postnatal day 35. Here, though, the length of social isolation was a major difference (2 weeks vs 8 weeks), raising the possibility that the result may still reflect the generation of new oligodendrocytes, but at the slower rate seen in adult mice (Dimou et al., 2008; Rivers et al., 2008; Young et al., 2013). Both studies are therefore consistent with activity‐dependent adaptation of myelin sheaths being most or only effective in differentiating and newly formed oligodendrocytes, with adaptive effects being substantially slower after developmental myelination simply because the rate of oligodendrocyte generation is less. Also consistent with the idea that myelin sheath number and properties are formed during a “critical window” soon after oligodendrocyte differentiation, live imaging studies in zebrafish and primary rodent oligodendrocyte–neuron co‐cultures have indicated that sheath numbers (one parameter under the influence of adaptive myelination) are set and established within a 5–6 h time window of an oligodendrocyte's life span (Watkins et al., 2008; Czopka et al., 2013). Furthermore, optogenetic stimulation of the adult mouse motor cortex results in an increase in oligodendrocyte precursor cells followed by an increase in myelin thickness and modified motor behavior, which can be prevented by the addition of histone deacetylase inhibitors that block the formation of new oligodendrocytes (Gibson et al., 2014). Evidence that new oligodendrocytes are important for learning—an assumed consequence of adaptive myelination—comes from the finding by the Richardson lab that newly formed adult oligodendrocytes enhance motor learning (McKenzie et al., 2014; Xiao et al., 2016). However, while this might suggest that new sheaths contribute to an adaptive myelination process that underpins learning (McKenzie et al., 2014), the very rapid emergence of differences in motor performance between control animals and those unable to form new oligodendrocytes (a few hours) would mean that any myelin formation responsible would need to be extremely rapid (Xiao et al., 2016). While this is certainly possible—oligodendrocytes complete initial sheath formation within 5–6 h (Watkins et al., 2008; Czopka et al., 2013)—it highlights the possibility that other functions of the oligodendrocyte that might not require the formation of a sheath such as metabolic support of the axon or the formation of prenodes (Kaplan et al., 2001, 1997; Fünfschilling et al., 2012; Lee et al., 2012b; Freeman et al., 2015) could also be responsible for the observed effect on motor learning.

In support of adaptation occurring after myelination is complete, transgenic overexpression experiments suggest that myelin sheath thickness and number can be increased in already mature oligodendrocytes. Activating Akt or MAPK signaling in differentiated oligodendrocytes engenders a degree of plasticity within already formed sheaths as evidenced by expansion of the leading edge of the inner myelin layer (the inner tongue), an increase in myelin thickness, or the addition of extra myelin sheaths (Goebbels et al., 2010; Snaidero et al., 2014; Jeffries et al., 2016). While each of these studies generates sustained signaling within the oligodendrocytes that is unlikely in a physiological situation, they do highlight the need to address whether already established oligodendrocytes and myelin sheaths can undergo modification in response to extrinsic cues.

## IS THE BALANCE BETWEEN THE INTRINSIC AND ADAPTIVE PATHWAYS THAT WILL SHAPE THE ULTIMATE MORPHOLOGY OF EACH OLIGODENDROCYTE THE SAME ACROSS DIFFERENT REGIONS OF THE CNS?

A question posed by a model in which oligodendrocyte morphology reflects the contributions of both intrinsic and adaptive pathways is whether the role of each is similar throughout the CNS. Different oligodendrocyte populations may have hard‐wired properties that not only dictate the number and size of myelin sheaths formed, but also how they respond to varying adaptive signals. Oligodendrocyte populations identified with single‐cell RNA sequencing show heterogeneity in expression of numerous ion channels, including glutamatergic channels (Marques et al., 2016), suggesting that different oligodendrocyte populations will have differential responses to neuronal activity. This will be interesting work for further study to understand how myelin adaptation occurs throughout the CNS. The most striking difference may be between white and gray matter oligodendrocytes. C^14^‐based turnover analyses in humans show that gray and white oligodendrocytes differ, with later expansion and greater turnover of the former population (Yeung et al., 2014). In rodents, differences between gray and white matter oligodendroglial cells were also demonstrated with transplantation studies (Viganò et al., 2013). Oligodendrocytes in the two regions are therefore behaviorally distinct and, given the role of gray matter oligodendrocytes in behaviors regulated by adaptive myelination and the intermittent myelin profile permitting de novo myelin sheath formation (Tomassy et al., 2014), we speculate that adaptive mechanisms will play a greater role in determining final oligodendrocyte morphology in gray matter. There is an intuitive logic to this in that the principal function of white matter tracts is rapid impulse conduction, as would be achieved by simply optimizing myelin sheath length and thickness in proportion to axon diameter, while in gray matter, a greater variation allows the alterations in circuit function required for more intricate connectivity resulting from activity‐dependent plasticity.

## CONCLUSIONS

The notion that myelination changes in response to activity was proposed nearly a century ago, and the recent confirmation by a variety of technologies is an important conceptual advance for the field. Allied to the demonstration of an intrinsic pathway able to sense microfiber diameter and generate myelin sheaths of physiological length around physical substrates of appropriate size and shape, a model emerges in which myelination, like synaptogenesis, is initially a hard‐wired process that is then sculpted by experience. The “smart wiring” so generated could play a significant role in learning, and manipulation of the two phases of myelination during live imaging of single oligodendrocytes combined with examination of behavior and learning will be required to test this. Another key area of further study will be the extent to which such a sequential model operates in the human CNS. Transcriptomic and proteomic studies show a number of differences between human and rodent oligodendrocytes and myelin (Ishii et al., 2009; Sim et al., 2009; Gopalakrishnan et al., 2012; Zhang et al., 2016). Do these differences also result in differing capacities for adaptive myelination between human and rodent cells? The answers will be important for our understanding of diseases of learning such as autism, in which adaptation might be impaired, and of adult diseases involving remyelination such as multiple sclerosis. In the latter case, we need to know whether myelin formed during regeneration shows adaptability or whether a loss of this contributes to the cognitive changes seen in this and other adult white matter diseases.
